# Impact of KNO_3_-Based Salt Nitriding Treatment on the Microstructure and Corrosion Resistance of Steel 20MnCr5 †

**DOI:** 10.3390/ma18081857

**Published:** 2025-04-18

**Authors:** Matej Fonović, Dario Kvrgić, Lovro Liverić, Ivna Kavre Piltaver

**Affiliations:** 1 Faculty of Engineering, University of Rijeka, Vukovarska ulica 58, 51000 Rijeka, Croatia; dario.kvrgic@riteh.uniri.hr; 2 Faculty of Engineering, Juraj Dobrila University of Pula, Nergrijeva ulica 6, 52100 Pula, Croatia; lliveric@unipu.hr; 3 Faculty of Physics, University of Rijeka, Ulica Radmile Matejčić 2, 51000 Rijeka, Croatia; ivna.kavre@uniri.hr

**Keywords:** steel, 20MnCr5, salt-bath nitriding, potassium nitrate, corrosion, hardness, nanoindentation

## Abstract

This study investigates the impact of KNO_3_-based salt bath nitriding on the microstructure, hardness, and corrosion resistance of 20MnCr5 steel. The nitriding process was conducted at 600 °C for 3 h and resulted in a nitrogen diffusion zone with a thickness that varied across the specimen, reaching a maximum of 70 μm. X-ray diffraction (XRD) analysis revealed no detectable nitrides, indicating nitrogen primarily occupied interstitial sites in the ferrite lattice and caused a lattice expansion of ~0.16%. Nanoindentation measurements showed an 80% increase in surface hardness (10.2 GPa) compared to the substrate (5.67 GPa), attributed to the solid solution strengthening mechanism. In contrast, however, an 18% decrease in Young’s modulus was observed near the surface, likely due to nitrogen-induced lattice distortions and crystal defects. Electrochemical tests in a 3.5 wt.% NaCl solution showed improved corrosion resistance, with the nitrided specimen exhibiting a 58% lower corrosion rate (1.275 mm/year) compared to untreated steel (3.04 mm/year). Despite a cathodic shift in corrosion potential, indicating localized susceptibility, the surface layer acted as a partial barrier to chloride ingress. The study demonstrates that KNO_3_-based salt nitriding is an environmentally friendly alternative to cyanide-based processes that offers good surface hardness and corrosion resistance, but needs to be further optimized.

## 1. Introduction

Corrosion, fatigue, creep, wear, and similar mechanisms are critical factors contributing to material degradation and failure in contemporary engineering systems. These processes typically arise from the mechanical and/or chemical interactions between engineering components and various environmental factors. It is estimated that the economic consequences of such degradation are substantial, with annual losses representing approximately 8% of a nation’s gross national product (GNP) [1]. Therefore, a deeper understanding of material deterioration and degradation mechanisms holds significant potential for resource savings, enabling the development of new innovative strategies to enhance various material properties.

The service life and performance of many engineering components, including machine parts, are predominantly influenced by their surface properties. To enhance these properties, various methods and techniques are employed, including nitriding a well-known thermochemical surface engineering technique [1]. This process modifies the surface of the treated metal, improving its mechanical, tribological, and corrosion-resistant characteristics. The improvement in these surface layer properties arises from alterations in chemical composition, which result in distinct microstructural characteristics. The primary mechanism of nitriding treatment involves the inward diffusion of atomic nitrogen into the surface-adjacent regions of various metallic substrates at elevated temperatures, typically ranging between 400 °C and 600 °C, resulting in the formation of a hardened surface layer [2,3].

The nitrogen is typically supplied through gaseous sources (commonly NH_3_, rarely N_2_), plasma (sometimes referred to as glow-discharge nitriding), or, less frequently, nitrogen-containing molten salts [1,4]. The salt bath nitriding is typically conducted at high temperatures using a molten salt bath, which often involves highly toxic cyanide salts such as KCN (potassium cyanide), NaCN (sodium cyanide), and similar chemical compounds [5,6]. Beyond its toxicity, the use of cyanide salt baths for nitriding poses considerable environmental and health risks [7]. To overcome these challenges, the salt bath nitriding can alternatively employ eco-friendly, non-toxic salts, predominantly composed of potassium nitrate (KNO_3_). The use of KNO_3_ salt as a nitrogen-donating medium has been documented in the literature for steels [8,9,10,11,12,13] and other metals [14,15,16]. The nitrogen source in the nitriding process is attributed to nitrous oxide (NO) and nitrogen dioxide (NO_2_), generated through the thermal decomposition of KNO_3_ at temperatures above 500 °C [17,18]. Moreover, these studies also suggest that these reactive nitrogen species, along with molecular nitrogen released during KNO_3_ decomposition at higher temperatures, actively participate in the nitriding process. This reactive nitrogen species facilitates inward diffusion into the surface-adjacent regions of the treated material, enabling the formation of a hardened nitrided layer and/or diffusion zone [19,20].

These investigations have reported significant improvements in the use of non-toxic salts for nitriding processes, demonstrating that these environmentally friendly alternatives can achieve comparable surface hardness and mechanical properties to traditional cyanide-based methods. Unlike traditional nitriding methods that use toxic cyanide-based salts, KNO_3_-based salts offer a safer and more sustainable alternative. The key benefits of this proposed substance are its affordability, lack of cyanides and toxic compounds, and minimized risk of adverse effects on the ecosystem. Despite this, there is still a lack of comprehensive data and knowledge on the practical use of eco-friendly, non-toxic salts in industrial applications. Moreover, a review of existing literature revealed a significant need to investigate the corrosion resistance of materials, particularly steel, treated using KNO_3_-based salts, as limited data are available on their performance in corrosive environments. To date, no studies have reported on the corrosion performance of such materials following treatment in KNO_3_-based salts.

In addition to the experimental corrosion assessment methods employed in this study, recent advances in computational techniques and artificial intelligence (AI) have emerged as complementary approaches for corrosion analysis. Finite element modeling (FEM) coupled with multi-physics simulations enables a comprehensive understanding of complex corrosion processes under dynamic and variable conditions [21]. Moreover, artificial intelligence approaches, notably convolutional neural networks (CNNs), have demonstrated significant potential in predicting corrosion damage and assessing corrosion risk with greater precision compared to traditional analytical methods [22]. While beyond the scope of the current experimental study, computational modeling and machine learning present compelling opportunities for the systematic optimization of surface treatment strategies for corrosion protection. Future studies could investigate the integration of physics-based modeling with AI-driven predictive frameworks to enhance mechanistic understanding of degradation processes and accelerate the development of tailored corrosion-resistant materials.

Therefore, the main objective of this study was to investigate the corrosion resistance, microstructure, and hardness properties of 20MnCr5 steel after a 3 h salt bath nitriding treatment in a KNO_3_-based solution at a temperature of 600 °C. The microstructure and morphology of the nitrided specimens were investigated by scanning electron microscopy (SEM) and optical microscopy (OM). The surface composition was analyzed by energy dispersive spectroscopy (EDS), while phase identification was performed by X-ray diffraction (XRD). In addition, the cross-sectional hardness of the thin surface layer was determined by nanoindentation measurements, while the surface hardness was determined by Vickers hardness tests. The electrochemical behavior of both the nitrided and unnitrided steel specimens was analyzed in a 3.5 wt.% NaCl solution using open circuit potential (OCP), linear polarization (LP), and electrochemical impedance spectroscopy (EIS). The analysis showed that the steel specimen nitrided in the KNO_3_-based solution exhibited significantly higher surface hardness and better electrochemical performance compared to the unnitrided steel specimen.

## 2. Materials and Experimental Procedure

### 2.1. Materials

Nitriding experiments were conducted on the 20MnCr5 steel specimens. This steel grade is a widely used case-hardening steel known for its excellent mechanical properties and ability to achieve high surface hardness through heat treatment. The advantages of this particular steel type include excellent hardenability and wear resistance after case hardening, good machinability in the annealed condition, and its suitability for complex geometries due to its combination of toughness and strength. The applications of this material include gears, shafts, and other components in the automotive industry, machine parts requiring high wear resistance and surface hardness, and components subjected to high stress and fatigue [23]. For example, a common practical application of this steel grade is to achieve a hard outer case surrounding a soft, tough core, effectively extending the lifespan of various engineering parts. This unique combination of properties makes this type of steel highly suitable for experimental studies and investigations. The steel 20MnCr5 used in this work was purchased from Strojopromet-Zagreb Ltd. (Zagreb, Croatia), a supplier of many industrial materials. The chemical composition of the steel prior to nitriding is experimentally determined using the glow discharge optical emission spectrometry (GDOES) characterization technique. The average results of the chemical composition analysis after five measurements are presented in Table 1.

**Figure 1:**
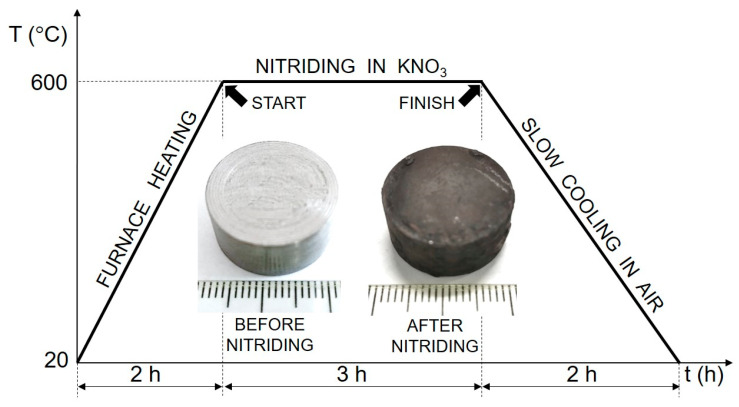
Complete thermal cycle of the nitriding treatment in a non-toxic mixture containing 80 wt.% KNO_3_ and 20 wt.% KCl, along with the appearance of the specimen before nitriding and after the treatment.

In order to prepare the specimens for nitriding treatment, a 25 mm diameter steel rod was sectioned into 10 mm thick segments (cf. Figure 1) using a circular diamond saw. To ensure uniform surface conditions and eliminate scratches or imperfections that could affect the nitriding process prior to the nitriding treatment, all specimens were subjected to a mechanical preparation procedure. The specimens were ground with silicon carbide sandpapers of progressively finer grits (#180, #320, #600, #800, #1200, #2000, and #4000) followed by final polishing using a 1-micrometer diamond paste to achieve a mirror-like surface finish. After surface preparation, all specimens were ultrasonically cleaned in ethanol for 10 min to remove residual surface contaminants.

### 2.2. Nitriding in Non-Toxic Salt Bath

Immediately following the cleaning step, the specimens underwent nitriding treatment using a KNO_3_-salt bath as the nitrogen-donating medium. A binary salt mixture composed of 80 wt.% KNO_3_ and 20 wt.% potassium chloride (KCl) was prepared in a glass beaker. To minimize the surface oxidation of the specimen, KCl was added to the non-toxic salt bath as a flux agent, as recommended in Ref. [24]. The salt mixture was then poured into a ceramic crucible and placed inside a laboratory-scale muffle furnace. The furnace was heated for 2 h until the salt mixture became fully molten. Afterward, the polished specimen was positioned at the bottom of the crucible to ensure full immersion in the molten salt bath during the nitriding process.

The nitriding treatment was performed at a temperature of 600 °C for a duration of 3 h. The nitriding temperature of 600 °C in a KNO_3_ salt bath is strategically chosen to harmonize thermodynamic viability, kinetic control, and industrial applicability. At this temperature, KNO_3_ undergoes complete decomposition, releasing reactive nitrogen species while preserving salt bath integrity and minimizing thermal degradation. The controlled nitrogen flux facilitates steady diffusion into the material’s subsurface, promoting the formation of a surface nitride layer. The full thermal cycle of nitriding treatment and specimen appearance before and after nitriding treatment are presented together in Figure 1. After the nitriding treatment, the specimen was removed from the crucible and air-cooled to room temperature. After the nitriding treatment, the specimen underwent ultrasonic cleaning in ethanol solution for 5 min to dislodge surface scale, i.e., primarily oxides and nitriding byproducts formed during the nitriding treatment. The ultrasonic bath ensured efficient removal of loosely adherent debris while preserving the integrity of the surface layer.

### 2.3. Materials Characterization

To examine and investigate the nitrided surface and diffusion zone, the specimen was cross-sectioned using a precision diamond saw. After sectioning, the specimen was embedded in epoxy resin to form cylindrical blocks, ensuring stability during metallographic examination. The mounted specimens were then subjected to sequential grinding with progressively finer silicon carbide sandpapers (from #180 to #4000 grit) and polished using a 1-micrometer diamond suspension to achieve the required surface finish for microscopic analysis. To prepare the specimens for metallographic inspection, they were chemically etched at room temperature for 15 s using a 4% Nital solution—a mixture of nitric acid and ethanol in an aqueous solution. This etching process selectively reveals the microstructure of the substrate and of the surface-adjacent area, enabling detailed metallographic analysis of the microstructure.

Basic metallographic analysis of the etched cross-sectional specimens was performed using an Olympus BX51 M optical microscope (Olympus, Tokyo, Japan) equipped with a digital imaging system. Subsequent metallographic analysis was conducted on a JSM-7800F (JEOL, Tokyo, Japan) scanning electron microscope (SEM), equipped with an energy-dispersive X-ray spectroscopy (EDS) detector (Oxford Instruments, Abingdon, UK) to investigate the microstructure of the surface-adjacent area, composition, and surface morphology in high-resolution detail.

Phase identification and analysis were conducted using a Rigaku Ultima IV X-ray diffractometer (Rigaku, Tokyo, Japan) in Bragg–Brentano geometry equipped with a Cu Kα radiation source (λ = 1.5405 Å). The measurement parameters for the X-ray diffraction (XRD) analysis were configured as follows: the scanning rate was 10.00°/min with a 0.020-degree step size. The scan range (2*θ*) was established between 30° and 90°. The obtained XRD data were analyzed using Rigaku software (SmartLab Studio II), and the recorded Bragg reflections were identified with the International Centre for Diffraction Data (JCPDS-ICDD) database [25].

The surface microhardness of the treated specimen was measured using a Vickers microhardness indenter (Zwick & Roell, Ulm, Germany) under a load of 98.07 N (HV 10) with a dwell time of 10 s at maximum load. A total of 10 indentations were performed on the surface of the nitrided specimen. The cross-sectional hardness measurements of the nitrided specimen were conducted using the nanoindentation technique. Due to the extremely thin nature of the diffusion zone, the conventional Vickers microhardness test method was unsuitable, particularly for measuring hardness as a function of depth. The nanoindentation investigations were conducted using a Nano Indenter G200 (Keysight Technologies, Santa Rosa, CA, USA) at room temperature. The nanoindentation measurements were systematically arranged in a 3 × 3 matrix, with 50 µm spacing between adjacent indents. This spacing was chosen to prevent overlapping plastic zones from neighboring indents, ensuring data accuracy and capturing representative hardness and Young’s modulus values across the nitrided cross-section area. The continuous stiffness measurement (CSM) technique was employed with 3 indents conducted near the surface of the treated material, 3 indents in the middle of the diffusion zone, and finally 3 indents in the substrate material. A Berkovich diamond indenter, characterized by its three-sided pyramidal shape and a total included angle of 142.3°, was used for the indentation measurements [26]. Prior to conducting the indentations, the specimen was meticulously polished using a polycrystalline diamond paste with a particle size of 1-micrometer to minimize surface damage and prevent dirt accumulation. In all nanoindentation tests, the drift rate was rigorously controlled to remain below 0.05 nm/s to reduce the adverse effects of temperature fluctuations and ensure more precise measurements. The indentations were performed using a maximum force of 500 mN, selected to achieve a maximum penetration depth of approximately 2000 nm. As the load is applied, the penetration depth is measured, and the load–displacement curve is automatically recorded for each indentation. The contact area at full load is determined based on the depth of the impression and the known angle or radius of the Berkovich indenter. The hardness values are calculated by dividing the applied load by the contact area, which is derived using the standard Oliver and Pharr method applied to the load–displacement curves [27]. All measurements, analyses, microstructural investigations, and characterization were conducted at room temperature.

### 2.4. Electrochemical Investigations

To evaluate the corrosion performance of the nitrided and unnitrided specimens, a series of electrochemical measurements were conducted using an Interface 1010E potentiostat (Gamry Instruments, Warminster, PA, USA). The measurements were carried out in a standard three-electrode electrochemical cell, consisting of the test specimen as the working electrode, a saturated Ag/AgCl reference electrode, and a graphite rod as the counter electrode. The electrolyte used was a 3.5 wt.% NaCl solution at room temperature. Before starting electrochemical tests, the specimens were immersed in the electrolyte for 60 min to achieve stabilization of the open circuit potential (OCP).

The open circuit potential (OCP) measurements were performed by continuously recording potential values over 60 min without external current application, ensuring the establishment of thermodynamic equilibrium prior to further electrochemical characterization [28].

Subsequently, the linear polarization resistance (LPR) measurements were performed by applying small potential perturbations of ±20 mV around the stabilized open circuit potential. The polarization resistance (Rp) was calculated from the slope of the resulting linear current-potential relationship and was inversely correlated to the instantaneous corrosion rate via the Stern–Geary equation [28].

Electrochemical impedance spectroscopy (EIS) was then employed to further evaluate the corrosion performance of the nitrided and unnitrided specimens. The EIS measurements were conducted at the stabilized open circuit potential, with spectra recorded over a frequency range of 100 kHz to 0.01 Hz using a sinusoidal voltage perturbation of 10 mV. In this technique, an alternating current (AC) signal of varying frequency is applied to the electrode system, and the resulting voltage response is measured to determine the impedance. The obtained impedance data were fitted to an equivalent circuit model to characterize the electrochemical behavior of the specimens, providing insights into charge transfer resistance, double-layer capacitance, and other interfacial properties relevant to corrosion processes [29].

Finally, the potentiodynamic polarization measurements were conducted to directly assess the electrochemical kinetics of the corrosion process. The tests were performed in a 3.5 wt.% NaCl solution by scanning the potential from −0.6 V to +2.5 V relative to the stabilized open circuit potential at a scanning rate of 2 mV/s. The resulting polarization curves provided key electrochemical parameters, including the corrosion potential (*E_corr_*), corrosion current density (*i_corr_*), and corrosion rate, which were determined using Tafel extrapolation [28]. All corrosion tests were carried out at ambient temperature. These measurements provide complementary insights to the electrochemical impedance spectroscopy and linear polarization resistance analyses, contributing to a more comprehensive understanding of the corrosion mechanisms and interfacial dynamics.

## 3. Results and Discussion

### 3.1. Microstructure and Morphology

**Figure 2:**
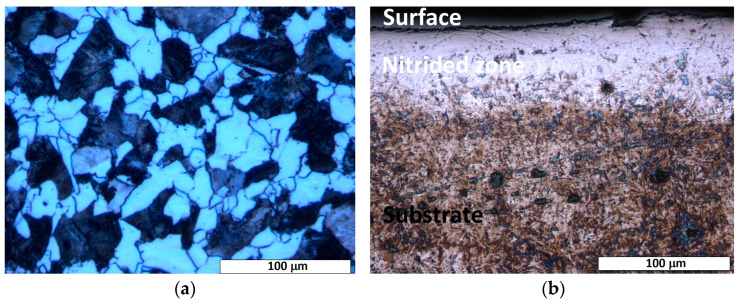
Light-optical micrograph of the etched cross-section of nitrided 20MnCr5 steel, illustrating: (**a**) a ferrite–pearlite microstructure, characteristic of the steel’s as-received state, and (**b**) two distinct microstructural regions: the substrate (base material) and the diffusion zone. A continuous nitrogen-rich surface layer, formed during nitriding, is evident at the interface, demarcating the transition between the bulk substrate and the nitrogen-enriched diffusion zone.

The etched microstructure of the as-received 20MnCr5 steel shows a ferrite–pearlite matrix with a fine-grained structure (cf. Figure 2a). The ferrite dominates the microstructure with a significantly higher volume fraction compared to pearlite. This is consistent with the chemical composition of the steel (cf. Table 1), where the low carbon content (<0.25 wt.%) favors ferrite formation. The microstructure shows evenly distributed pearlite colonies within the ferrite matrix, which are characterized by their well-known lamellar structure (ferrite and cementite layers).

**Figure 3:**
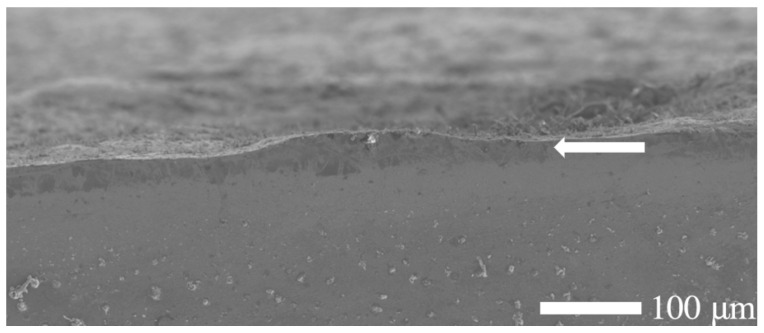
Scanning electron microscopy (SEM) micrograph of the etched cross-section of a nitrided 20MnCr5 steel specimen, revealing a distinguishable yet thinner and non-uniform diffusion zone near the surface. Note that the treated surface is rough, and defects such as pores and micro-cracks might exist on the plain surface.

Cross-sectional optical microscopy of the nitrided specimen revealed a diffusion zone with non-uniform thickness, extending up to a maximum of 70 μm across the sample (cf. Figure 2b). As shown in Figure 2b, a distinguishable etching contrast is evident between the surface-adjacent diffusion zone and the substrate material, highlighting their microstructural differences and different corrosion behavior. The diffusion zone shows a brighter etching contrast, possibly due to nitrogen saturation and/or nitride phase formation. While a continuous diffusion zone can be observed in most regions (cf. Figure 2b), local thinning (cf. Figure 3) or the absence of the diffusion zone can be seen in certain areas of the treated steel specimen. This heterogeneity indicates possible variations in the nitrogen diffusion kinetics or in the process conditions (e.g., temperature gradients, surface impurities, or uneven contact with the salt bath surface). In the areas where the nitrided layer is compact, there is no significant evidence of cracks, voids, or interfacial decohesion, indicating robust adhesion and structural integrity (cf. Figure 2b and Figure 3).

In contrast, the substrate (core of the specimen) remained unaffected, preserving the steel’s bulk microstructure and morphology. During the salt bath nitriding process with non-toxic KNO_3_, oxide layers form on the specimen’s surface due to the high oxidizing environment created by the thermal decomposition of KNO_3_ at high temperatures (>500 °C) [30]. Consequently, a minor amount of residual surface oxide is visible along the edge of the nitrided specimen, as illustrated in Figure 1. In addition, the SEM analysis (cf. Figure 3) shows an uneven and rough surface of the nitrided specimen, which indicates possible microstructural defects of the surface, such as pores, voids, and microcracks.

In addition, some studies report the formation of a white layer (a compound layer rich in ε-Fe_2-3_N or γ’-Fe_4_N nitrides) on the surface of nitrided specimens [11,12]; however, such a layer is not present in this study, as only the diffusion zone is clearly visible.

### 3.2. Phase Analysis

**Figure 4:**
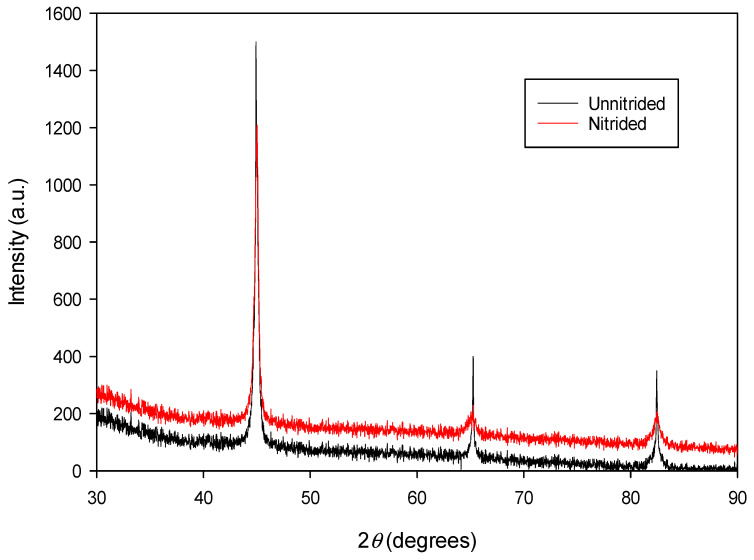
The XRD spectrum of unnitrided 20MnCr5 steel specimen (red line) compared with that of the steel specimen nitrided in a non-toxic salt bath at 600 °C for 3 h (black line).

The diffractogram of the nitrided specimen (cf. Figure 4) confirms the presence of a ferritic matrix (α-Fe), consistent with the original material (cf. Figure 4). Distinct *α*-Fe peaks corresponding to (100), (200), and (211) crystallographic planes are observed, consistent with Bragg’s law. At the surface of the nitrided specimen, no peaks corresponding to nitride phases (e.g., ε-Fe_2-3_N, γ’-Fe_4_N, CrN, etc.) were detected within the instrument’s sensitivity limits. This suggests that either no nitrides are present, the nitride concentration falls below the detection threshold of the conventional XRD method, or, in rare cases, the nitrides may exist in an amorphous or ultra-fine crystalline form that cannot be resolved by this technique.

A detailed examination of the XRD data, including peak fitting routine using a pseudo-Voigt function, revealed that the lattice parameters of the nitrided 20MnCr5 material—calculated from the d-spacings of their respective (hkl) lattice planes—are consistently larger than those of the untreated substrate. A comparative summary of the estimated lattice parameters for the nitrided and as-received (unnitrided) 20MnCr5 steel is provided in Table 2.

A comparison of the lattice parameters presented in Table 2 indicates that the estimated lattice parameters of the nitrided 20MnCr5 material are systematically higher than those of the unnitrided sample. This observation suggests that atomic nitrogen, diffused into the α-Fe matrix during nitriding, predominantly occupies interstitial sites within the body-centered cubic iron lattice, inducing nitrogen-driven lattice expansion. The average increase in lattice parameters across the three analyzed pairs is ~0.16%, as determined by the XRD analysis. This subtle yet statistically significant expansion correlates with nitrogen interstitial incorporation into the body-centered cubic (BCC) lattice during nitriding, consistent with the comparative analysis presented in Table 2. The low increase in lattice parameters (~0.16%) observed in the nitrided sample suggests a relatively low nitrogen concentration dissolved in the α-Fe matrix. This aligns with the absence of nitride phase peaks (e.g., ε-Fe_2-3_N, γ’-Fe_4_N) in the XRD analysis (cf. Figure 4), which indicates that nitrogen atoms primarily occupy interstitial sites in the BCC lattice rather than forming distinct nitride precipitates. The minimal lattice expansion aligns with the expected low nitrogen concentration, favoring solidsolution strengthening over nitride phase formation. The surface nitrogen concentration, measured using the EDX method, corresponds to approximately 0.8 wt.%. While nitrogen uptake typically causes residual stresses in the material due to lattice expansion [4,31], the minimal increase in the lattice parameter observed in this sample (~0.16%) indicates that no significant residual compressive stresses have likely developed. This is further supported by the absence of large cracks, voids, or interfacial decohesion in the nitrided layer (cf. Section 3.1). A comparable increase in the lattice parameters of the nitrided specimen, as observed in this study, has been documented and analyzed in Ref. [10], where the incorporation of nitrogen interstitials into the BCC lattice was identified as the primary cause of lattice expansion. The reported increase in lattice parameters is approximately 0.08%.

### 3.3. Hardness Measurements

The unnitrided material exhibits a surface hardness of 255 HV 10, as measured by standard Vickers microhardness testing equipment. In contrast, the nitrided specimen exhibits a significant increase in surface hardness, ranging from 300 HV 10 to 625 HV 10. This gradient in hardness aligns well with the observed variations in the thickness of the nitrided layer across the sample surface, as discussed in Section 3.1.

**Figure 5:**
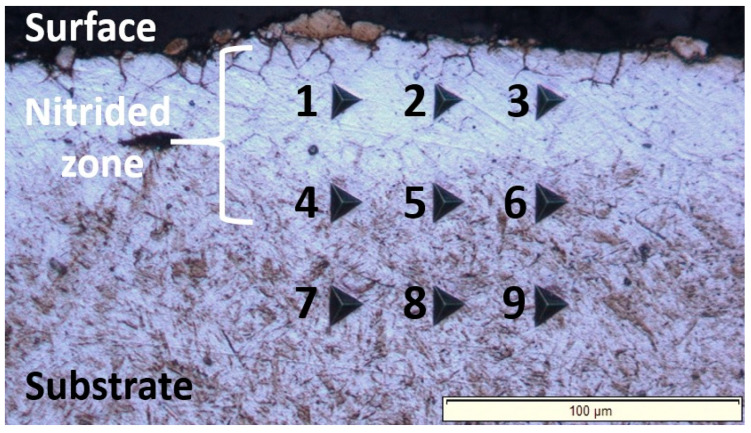
Light-optical micrograph obtained through the nanoindentation measurements carried out across the cross-section of the nitrided 20MnCr5 steel, covering both the surface and the substrate of the treated material. The numbered indents (1–9) correspond to the nanoindentation data compiled in Table 3, enabling a direct comparison of hardness and Young’s modulus across the cross-section. Indents 1–3 are located in the near-surface region; indents 4–6 are positioned in the intermediate zone, and indents 7–9 are situated in the substrate (untreated ferrite–pearlite microstructure).

The nanoindentation investigations revealed that the nitrided zone exhibits a graded hardness profile across its cross-section, with hardness values decreasing progressively from the surface-adjacent area to the substrate. This hardness gradient (nanoindentation measurement grid shown in Figure 5 and results presented in Table 3) correlates with the diminishing nitrogen concentration with depth [32,33]. The surface region achieves peak hardness, measuring approximately 1.8 times that of the untreated substrate, due to a solid solution hardening mechanism, i.e., the formation of an interstitial solid solution of nitrogen in the α-Fe crystal lattice. Summarized, the gradual reduction in hardness towards the core reflects the diminishing nitrogen content, ultimately approaching the baseline properties of the unmodified core material.

Additional determined data reveal an inverse correlation between Young’s modulus and hardness, with modulus values decreasing as hardness increases (cf. Table 3). This behavior contrasts with conventional trends in homogeneous materials, where hardness and stiffness often scale proportionally [34]. The observed anomaly may stem from microstructural heterogeneities introduced during nitriding, such as lattice distortions or residual stresses, which could decouple the typical hardness–Young’s modulus relationship [35]. Nitrogen interstitials disrupt the regular metal atomic arrangement, reducing the stiffness of atomic bonds locally. This lowers the material’s elastic response, i.e., Young’s modulus. Moreover, small amounts of nitrogen-induced crystal defects (e.g., vacancies, dislocations, stacking faults, etc.) can reduce the overall stiffness of the treated material [36]. The nitriding treatment could introduce compressive residual stresses near the surface-adjacent area, and this can alter the elastic recovery during indentation measurements, leading to a lower apparent Young’s modulus [37]. These three mechanisms: (i) lattice distortions, (ii) defect proliferation, and (iii) residual stress jointly disrupt the hardness–Young’s modulus relationship, leading to deviations from classical material behavior, as observed in this study.

### 3.4. Electrochemical Analysis

#### 3.4.1. Open Circuit Potential (OCP)

**Figure 6:**
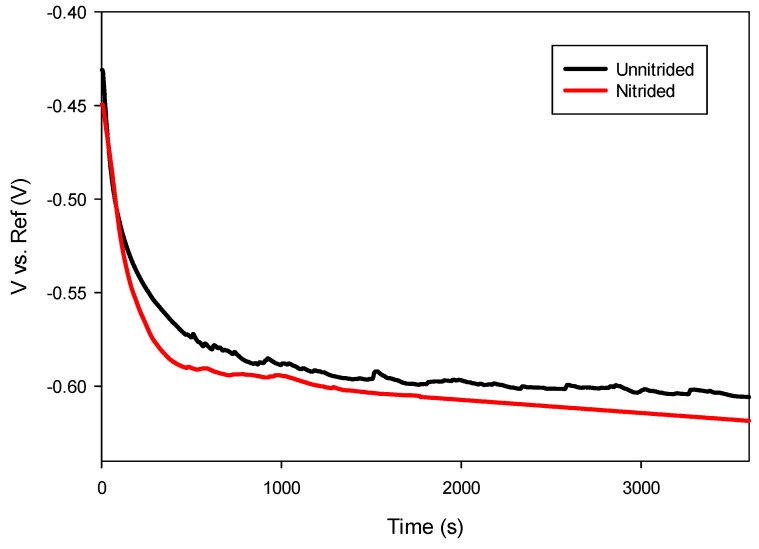
Open circuit potential measurements of unnitrided and nitrided 20MnCr5 steel specimens.

After immersion in the electrolyte, an initial decrease in potential was observed in both specimens during the first 10 min, attributed to the initiation of corrosion processes as the prepared surfaces interacted with the electrolyte. After approximately 30 min, the potential stabilized, indicating that thermodynamic equilibrium was achieved between anodic and cathodic reactions at the electrode–electrolyte interface [28]. As presented in Figure 6, the nitrided sample exhibited a slightly more negative open circuit potential compared to the unnitrided sample, suggesting reduced thermodynamic stability and increased susceptibility to anodic dissolution.

Generally, nitrided steels reported in the literature exhibit a shift toward more positive potentials due to the formation of stable nitride phases that enhance surface passivity [37,38]. However, the negative shift observed in this study, as presented in Table 4, suggests the presence of surface defects within the nitrided layer, promoting anodic activity and reducing passivation effectiveness.

#### 3.4.2. Linear Polarization Resistance (LPR)

Table 4 summarizes the LPR measurement results. The nitrided 20MnCr5 steel exhibited notably lower polarization resistance (R_p_) compared to the unnitrated specimen. The reduced R_p_ value for the nitrided sample indicates increased corrosion current density near the equilibrium potential, highlighting its decreased resistance to uniform corrosion.

Typically, nitrided surfaces provide enhanced corrosion protection through dense, adherent nitrided layers. However, the decreased R_p_ observed in this study suggests that microstructural defects, such as pores or micro-cracks within the diffusion zone, facilitated electrolyte penetration, thereby accelerating corrosion reactions [39].

#### 3.4.3. Electrochemical Impedance Spectroscopy (EIS)

Electrochemical impedance spectroscopy (EIS) was employed to provide a detailed analysis of corrosion processes occurring at the steel surface–electrolyte interface. This method allows insight into interfacial properties and the effectiveness of protective surface films by evaluating the electrical response of the specimens to small alternating current perturbations. The representative EIS spectrum for nitrided and unnitrided steel specimens immersed in a 3.5 wt.% NaCl solution was recorded and analyzed using equivalent circuit modeling [40,41].

**Figure 7:**
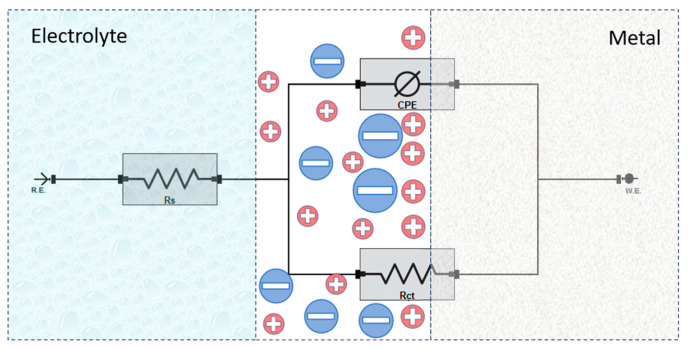
Equivalent circuit model used for fitting the EIS data.

To analyze the impedance response, an equivalent electrical circuit was employed to model the electrochemical behavior at the steel–electrolyte interface (cf. Figure 7). The circuit consists of solution resistance (R_s_), charge transfer resistance (R_ct_), and a constant phase element (CPE) representing the double-layer capacitance [40,41]. In this analysis, the charge transfer resistance (R_ct_) is considered equivalent to the polarization resistance (R_p_), as no additional resistive elements significantly contribute to the overall impedance response.

The key parameters obtained from the fitting of the EIS data are summarized in Table 5.

The unnitrided specimen exhibited relatively low polarization resistance, indicating active corrosion behavior typical for untreated low-alloy steels exposed to chloride-rich environments. Additionally, a high double-layer capacitance and a pronounced deviation from ideal capacitive behavior, which typically indicates the presence of microstructural irregularities or surface heterogeneity, suggest the existence of roughness, porosity, or other defects at the surface of the specimen. These observations align with the findings presented in Figure 3 and discussed in Section 3.1. The slightly elevated measured solution resistance compared to standard 3.5% NaCl solutions is attributed to geometric or cell-specific factors within the experimental setup [39].

**Figure 8:**
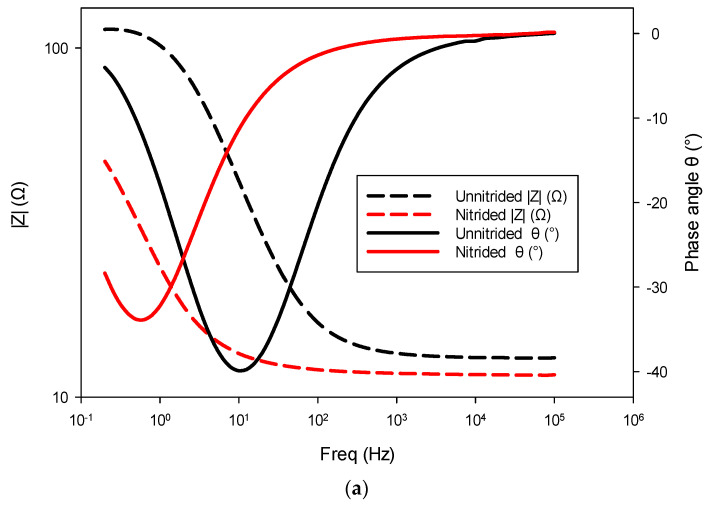

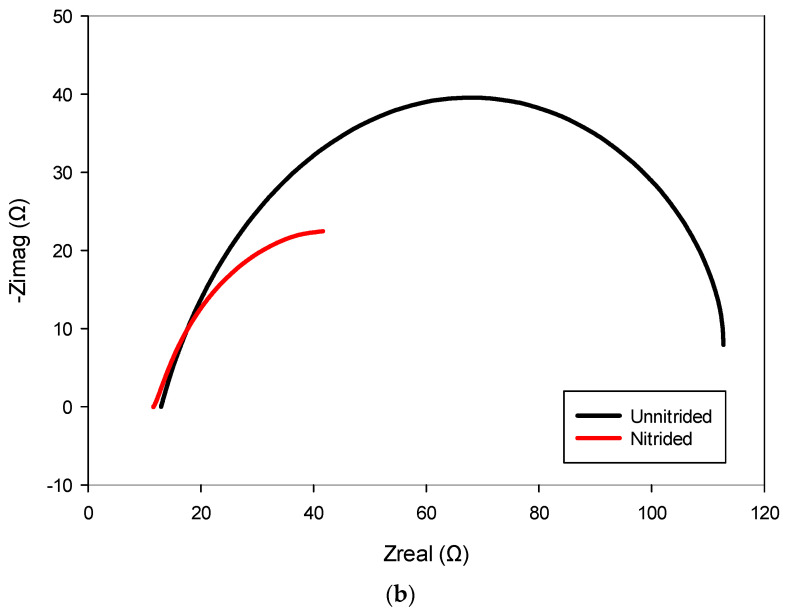
Electrochemical impedance spectroscopy (EIS) results for nitrided and unnitrided steel specimens: (**a**) Bode plot, and (**b**) Nyquist plot. Note that in the Bode plot (**a**), dashed lines represent the impedance magnitude (|Z|, Ω) axis, while solid lines correspond to the phase angle (θ, °) axis.

The nitrided specimen demonstrated notably reduced polarization resistance, indicating increased corrosion susceptibility compared to the untreated material. This marked reduction in R_ct_ suggests that the surface (nitrided) layer, rather than providing effective corrosion protection, introduced defects or porous pathways. Consistent with this observation, the nitrided specimen exhibited significantly higher double-layer capacitance, implying a larger electrochemically active surface area due to defects like micro-cracks and pores at the surface (cf. Figure 3). These trends are also evident in the impedance spectra, as observed in the Bode and Nyquist plots, where the nitrided specimen exhibits lower impedance and altered phase characteristics compared to the unnitrided steel specimen (cf. Figure 8a,b). Collectively, these electrochemical indicators clearly reveal that the nitriding process, under the studied conditions, produced a defect-rich nitrided layer, resulting in diminished protective effectiveness against corrosion [39,40,41].

#### 3.4.4. Potentiodynamic Polarization

**Figure 9:**
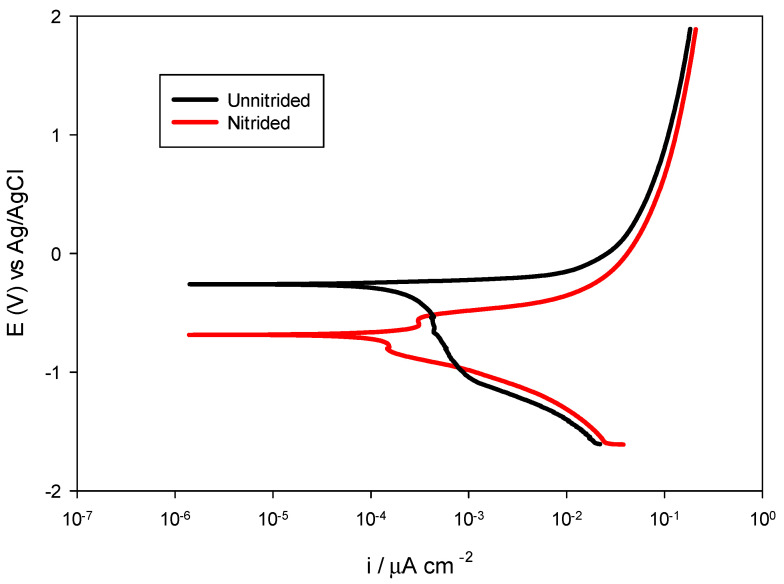
Potentiodynamic polarization curves for nitrided vs. unnitrided 20MnCr5 steel specimens.

The potentiodynamic polarization testing was performed to further elucidate the corrosion behavior of nitrided and unnitrided steel in a 3.5 wt.% NaCl solution. By applying a controlled potential sweep from −0.6 V to +2.5 V relative to the stabilized OCP at a scan rate of 2 mV/s, anodic and cathodic polarization curves were obtained, providing insight into the corrosion potential (*E_corr_*), corrosion current density (*i_corr_*), and corrosion rate of both materials (cf. Figure 8). These parameters, derived via Tafel extrapolation, allow for a comparative assessment of the corrosion resistance of the tested specimens under dynamic polarization conditions [28]. The results are summarized in Table 6 and presented in the Figure 9.

The unnitrided steel sample exhibited a corrosion potential of approximately −0.2594 V, accompanied by a relatively high corrosion current density of 130.3 μA/cm^2^ and an associated corrosion rate of 3.040 mm/year, indicative of pronounced uniform corrosion susceptibility in chloride-containing media.

The nitrided sample displayed a notably more negative corrosion potential of −0.6851 V, highlighting its increased thermodynamic susceptibility to corrosion initiation compared to the original, unnitrided steel specimen.

However, despite this negative shift, the nitrided specimen demonstrated significantly reduced corrosion kinetics, with a corrosion current density of only 54.66 μA/cm^2^ and a lower corrosion rate of 1.275 mm/year—approximately 58% lower than the non-nitrided sample. This seemingly contradictory finding, negative corrosion potential paired with reduced corrosion rate, can be explained by the localized corrosion phenomena: microstructural defects, such as pores and micro-cracks within the nitrided layer, facilitate anodic reactions locally, shifting *E_corr_* negatively, while the overall intact nitrided layer provides a substantial barrier effect, reducing the overall corrosion current [39].

This interpretation is supported by previous results of the OCP, LPR, and EIS analyses, which overall indicate surface heterogeneities and partial protection by the nitrided layer on the surface of the specimen.

Overall, the comprehensive electrochemical investigation with OCP, LPR, EIS, and potentiodynamic polarization revealed a complex corrosion behavior in the nitrided 20MnCr5 steel specimen. Although nitriding introduced surface defects that increased localized corrosion susceptibility, the presence of intact nitrided areas significantly reduced the overall corrosion kinetics.

## 4. Conclusions

Salt bath nitriding treatment of 20MnCr5 steel was successfully performed with a non-toxic KNO3-based salt mixture at 600 °C for 3 h. A comprehensive analysis and characterization of the treated specimens revealed the following important findings:

1. A diffusion zone of varying thickness was observed extending to a maximum depth of 70 μm throughout the sample. Although it was continuous in most areas, localized thinning or complete absence of the zone was observed in certain areas. The treated surface exhibits roughness, where defects such as pores and microcracks may be present.
2. X-ray diffraction analysis showed no detectable ε-Fe_2-3_N, γ’-Fe_4_N, or CrN phases, suggesting that nitrogen primarily occupies interstitial sites within the α-Fe lattice. This interstitial solid solution caused a measurable lattice expansion of 0.16%, confirming the effective dissolution and diffusion of nitrogen into the ferritic matrix material. The absence of distinct nitride phases suggests a diffusion-controlled mechanism driven by solid solution strengthening rather than compound layer formation.
3. The nanoindentation measurements revealed an 80% increase in surface hardness (10.2 GPa compared to 5.67 GPa in the substrate), which can be attributed to a mechanism of solid-state solidification by nitrogen interstitials. The Young’s modulus anomaly, i.e., a counterintuitive 18% reduction in Young’s modulus near the surface, was observed, which is likely due to nitrogen-induced lattice distortions, defect proliferation, and possible residual stresses that have disturbed the conventional hardness–stiffness relationship.
4. Improved corrosion performance: Despite a cathodic shift in corrosion potential (indicating greater thermodynamic susceptibility), the nitrided layer showed a 58% lower corrosion rate (1.275 mm/year) compared to untreated steel (3.04 mm/year) in 3.5% NaCl. This improvement is attributed to the nitrided layer acting as a partial barrier against chloride ingress, although corrosion is localized at microstructural defects (e.g., pores and microcracks), resulting in lower polarization resistance but lower overall corrosion current density.
5. Environmental and practical impact: The KNO_3_-based nitriding process has been demonstrated to be a non-toxic, environmentally friendly alternative to conventional cyanide-based nitriding processes, with high surface hardness and excellent corrosion resistance. Although the process has a high industrial profitability, further optimization of critical parameters such as temperature and treatment time is required to reduce surface defects (e.g., porosity, microcracks, thickness, etc.) and improve corrosion resistance for large components.

## Figures and Tables

**Table 1:** Experimentally determined chemical composition of the purchased 20MnCr5 steel.

Chemical Element	C	Si	Mn	S	Cr	Mo	Cu	Al	Ti	Fe
Content, wt.%	0.22	0.26	1.01	0.013	1.0	0.096	0.17	0.014	0.027	97.2
RSD, %	2.72	1.10	0.40	4.46	0.44	5.15	0.61	3.40	2.71	1.59

**Table 2:** An overview of the estimated lattice parameters of nitrided and unnitrided steel specimens.

Bragg Reflection	Nitrided Material		Unnitrided Material	
	*d* (Å)	*a* (Å)	*d* (Å)	*a* (Å)
(110)	2.0155	2.8503	2.0145	2.8489
(200)	1.4304	2.8608	1.4285	2.8570
(211)	1.1694	2.8644	1.1659	2.8558
Average lattice parameters		2.8585 Å		2.8539 Å

**Table 3:** Summary of estimated hardness properties (GPa) and Young’s modulus (GPa) values measured at different locations across the nitrided cross-section (from surface to substrate), as illustrated in Figure 5.

	Surface			Intermediate			Substrate		
Indent, location	1	2	3	4	5	6	7	8	9
Hardness, [GPa]	10.16	10.24	9.5	8.44	7.89	8.5	7.33	5.67	6.70
Young’s modulus, [GPa]	204	199	192	219	218	232	246	238	254

**Table 4:** Open circuit potential (OCP) and linear polarization (LP) results.

Sample	OCP [V]	R_p_ [Ω cm^2^]
Unnitraded	−0.6062	130.80
Nitraded	−0.6184	76.60

**Table 5:** Electrochemical impedance spectroscopy (EIS) results for nitrided and unnitrided 20MnCr5 steel specimens.

Specimen	Solution Resistance (Rs) [Ω]	Charge Transfer Resistance (Rct) [Ω]	CPE (Y_0_) [S·s^n^]	CPE Exponent (n)
Unnitraded	12.98	107.6	9.73 × 10^−4^	0.794
Nitraded	11.67	70.86	1.50 × 10^−2^	0.747

**Table 6:** Potentiodynamic polarization results for unnitrided and nitrided specimens.

Specimen	Corrosion Potential (*E_corr_*) [V]	Corrosion Current Density (*i_corr_*) [μA/cm^2^]	Corrosion Rate [mm/year]
Unnitrided	−0.2594	130.3	3.04
Nitrided	−0.6851	54.66	1.275

## Data Availability

All data are contained within the article. Further inquiries can be directed to the corresponding author due to institutional policy.

## References

[1] Mittemeijer E.J., Somers M.A.J. (2015). Thermochemical Surface Engineering of Steels.

[2] Fonović M. (2015). Nitriding Behaviour of Ni and Ni-Based Binary Alloys. Ph.D. Thesis.

[3] Mittemeijer E.J., Dossett J., Totten G.E. (2013). Nitriding and Nitrocarburizing of Steels. ASM Handbook: Steel Heat Treating Fundamentals and Processes.

[4] Dong H. (2010). S-phase surface engineering of Fe-Cr, Co-Cr and Ni-Cr alloys. Int. Mater. Rev..

[5] Funatani K. (2004). Low-Temperature Salt Bath Nitriding of Steels. Heat Treat. Met..

[6] Huzar T.F., George T., Cross J.M. (2013). Carbon monoxide and cyanide toxicity: Etiology, pathophysiology and treatment in inhalation injury. Expert Rev. Respir. Med..

[7] Bell T., Dearnley A. (1994). Environmental issues in surface engineering and related industrial sectors. Surf. Eng..

[8] Shen Y.Z., Oh K.H., Lee D.N. (2006). Nitriding of Interstitial Free Steel in Potassium–Nitrate Salt Bath. ISIJ Int..

[9] Hamdy A.S., Marx B., Butt D. (2011). Electrochemical studies on the film formed by direct nitridation of AA2024 in a KNO_3_ salt bath at low temperature. Mater. Chem. Phys..

[10] Shen Y.Z., Oh K.H., Lee D.N. (2005). Nitriding of steel in potassium nitrate salt bath. Scr. Mater..

[11] Bonow V.T., Maciel D.S., Fenner N.L., Reguly A., Zimmer A., Zimmer C.G. (2021). Nitriding in non-toxic salts bath: An approach to implement cleaner production in the metallurgic industry. Clean. Eng. Technol..

[12] Bonow V.T., Maciel D.S., Zimmer A., Zimmer C.G. (2019). Nitriding in low carbon steels using non-toxic salt baths. Rev. Lib..

[13] Lee T.H., Oh C.S., Lee M.K., Han S.W. (2010). Nitride precipitation in salt-bath nitrided interstitial-free steel. Mater. Charact..

[14] Zhu Y.S., Wei X.N., Yin Y.X. (2021). A novel low temperature and green salt bath nitriding of titanium alloy. Surf. Eng..

[15] Roy A., Shiva Kumar K., Raghunath A., Sharma R.C., Shekhar R. (2012). Feasibility and kinetics of nitriding of pure titanium and Ti–6Al–4V in molten salt bath of potassium nitrate. Surf. Eng..

[16] Sabuz E.H., Maruf M.A., Haider W., Shabib I. (2023). Enhanced Corrosion Resistance of TiZrN-Coated Additively Manufactured 8620 Low-Alloy Steel in Nitrate Salt Solution and Salt Bath. Coatings.

[17] Freeman E.S. (1957). The Kinetics of the Thermal Decomposition of Potassium Nitrate and of the Reaction between Potassium Nitrite and Oxygen. J. Am. Chem. Soc..

[18] Stern K.H. (1972). High Temperature Properties and Decomposition of Inorganic Salts Part 3, Nitrates and Nitrites. J. Phys. Chem. Ref. Data.

[19] Stein J., Schacherl R.E., Jung M., Meka S., Rheingans B., Mittemeijer E.J. (2013). Solubility of nitrogen in ferrite; the Fe–N phase diagram. Int. J. Mater. Res..

[20] Mittemeijer E.J., Somers M.A.J. (1997). Thermodynamics, kinetics, and process control of nitriding. Surf. Eng..

[21] Wang C., Xu S., Li W., Wang Y., Shen G., Wang S. (2024). Multi-physics coupled simulation and experimental investigation of alternating stray current corrosion of buried gas pipeline adjacent to rail transit system. Mater. Des..

[22] Wang C., Li W., Wang Y., Yang X., Xu S. (2019). Predictive model for corrosion hazard of buried metallic structure caused by stray current in the subway. Anti-Corros. Methods Mater..

[23] Brnic J., Turkalj G., Lanc D., Canadija M., Brcic M., Vukelic G. (2015). Comparison of material properties: Steel 20MnCr5 and similar steels. J. Constr. Steel Res..

[24] Lee M.K., Kim D.S., Kim S.C., Han S.W., Kim I., Lee D.N. (2010). Effect of NaCl and CaCl_2_ additives on NaNO_3_ bath nitriding of steel. Mater. Sci. Eng. A.

[25] The International Centre for Diffraction Data. https://www.icdd.com/.

[26] Riester L., Bridge R.J., Breder K. (1998). Characterization of Vickers, Berkovich, Spherical and Cube Cornered Diamond Indenters by Nanoindentation and SFM. Mater. Res. Soc. Symp. Proc..

[27] Oliver W.C., Pharr G.M. (1992). An improved technique for determining hardness and elastic modulus using load and displacement sensing indentation experiments. J. Mater. Res..

[28] Gamry Instruments Getting Started with Electrochemical Corrosion Measurement. Gamry Instruments, Inc.. https://www.gamry.com/assets/Application-Notes/Getting-Started-with-Electrochemical-Corrosion-Measurement.pdf.

[29] Gamry Instruments Basics of Electrochemical Impedance Spectroscopy. Gamry Instruments, Inc.. https://www.gamry.com/application-notes/EIS/basics-of-electrochemical-impedance-spectroscopy/.

[30] Shi P., Leng B., Ye X., Wang S., Chang L., Li X., Li X., Huang H. (2023). Tribological behavior of 316H stainless steel in NaNO_3_-KNO_3_ molten salt at elevated temperature. Sol. Energy Mater. Sol. Cells.

[31] Fonović M., Kvrgić D., Iljkić D., Liverić L. Gaseous nitriding of pure nickel—Observation and interpretation of microstructural features. Proceedings of the 12th International Conference Mechanical Technologies and Structural Materials.

[32] Nakada N., Tsuboi K., Onomoto T., Tsuchiyama T., Takaki S., Inden G. (2014). Thermodynamics and kinetics of solution nitriding. Calphad.

[33] Fonović M., Liverić L., Pavlović L., Jurković Z. Nitriding of steel 20MnCr5 using an environmentally friendly salt. Proceedings of the 13th International Conference Mechanical Technologies and Structural Materials.

[34] Cheng Y.T., Cheng C.M. (1998). Relationships between hardness, elastic modulus, and the work of indentation. Appl. Phys. Lett..

[35] Tromas C., Stinville J.C., Templier C., Villechaise P. (2012). Hardness and elastic modulus gradients in plasma-nitrided 316L polycrystalline stainless steel investigated by nanoindentation tomography. Acta Mater..

[36] Kharouji H., Dezerald L., Hirel P., Carrez P., Cordier P., Taupin V., Guénolé J. (2024). Atomistic to continuum mechanics description of crystal defects with dislocation density fields: Application to dislocations and grain boundaries. Int. J. Plast..

[37] Pharr G.M., Tsui T.Y., Bolshakovm A., Oliver W.C. (1994). Effects of Residual Stress on the Measurement of Hardness and Elastic Modulus using Nanoindentation. MRS Proc..

[38] Srikanth S., Pandurangan S., Joseph A., Ravi K. (2013). Surface Modification of Commercial Low-Carbon Steel using Glow Discharge Nitrogen Plasma and its Characterization. J. Mater. Eng. Perform..

[39] Gontijo L.C., Machado R., Kuri S.E., Casteletti L.C., Nascente P.A.P. (2006). Corrosion resistance of the layers formed on the surface of plasma-nitrided AISI 304L steel. Thin Solid Films.

[40] Herrera Hernández H., Ruiz Reynoso A.M., Trinidad González J.C., González Morán C.O., Miranda Hernández J.G., Mandujano Ruiz A., Morales Hernández J., Orozco Cruz R. (2020). Electrochemical Impedance Spectroscopy (EIS): A Review Study of Basic Aspects of the Corrosion Mechanism Applied to Steels. Electrochemical Impedance Spectroscopy.

[41] Tandon V., Patil A.P., Rathod R.C. (2020). Influence of Time on Low Temperature Salt Bath Nitriding and its Corrosion Behavior of 316L ASS in PEMFC Environment. Met. Phys. Chem. Surf..

